# Evaluation of metabolites in Iranian Licorice accessions under salinity stress and *Azotobacter* sp. inoculation

**DOI:** 10.1038/s41598-022-20366-6

**Published:** 2022-09-23

**Authors:** Seyyed Sasan Mousavi, Akbar Karami, Mohammad Jamal Saharkhiz, Mohammad Etemadi, Mohammad Mehdi Zarshenas

**Affiliations:** 1 Department of Horticultural Science, School of Agriculture, Shiraz University, Shiraz, 71441-13131 Iran; 2 Medicinal Plants Processing Research Center, Shiraz University of Medical Sciences, Shiraz, Iran; 3 Department of Traditional Pharmacy, School of Pharmacy, Shiraz University of Medical Sciences, Shiraz, Iran

**Keywords:** Biochemistry, Plant sciences

## Abstract

Licorice (*Glycyrrhiza glabra* L.) is an industrial medicinal plant that is potentially threatened by extinction. In this study, the effects of salinity (0 and 200 mM sodium chloride (NaCl)) and *Azotobacter* inoculation were evaluated on 16 licorice accessions. The results showed that salinity significantly reduced the fresh and dry biomass (FW and DW, respectively) of roots, compared to plants of the control group (a decrease of 15.92% and 17.26%, respectively). As a result of bacterial inoculation, the total sugar content of roots increased by 21.56% when salinity was applied, but increased by 14.01% without salinity. Salinity stress increased the content of glycyrrhizic acid (GA), phenols, and flavonoids in licorice roots by 104.6%, 117.2%, and 56.3%, respectively. Integrated bacterial inoculation and salt stress significantly increased the GA content in the accessions. Bajgah and Sepidan accessions had the highest GA contents (96.26 and 83.17 mg/g DW, respectively), while Eghlid accession had the lowest (41.98 mg/g DW). With the bacterial application, the maximum amounts of glabridin were obtained in Kashmar and Kermanshah accessions (2.04 and 1.98 mg/g DW, respectively). Bajgah and Kashmar accessions had higher amounts of rutin in their aerial parts (6.11 and 9.48 mg/g DW, respectively) when their roots were uninoculated. In conclusion, these results can assist in selecting promising licorice accessions for cultivation in harsh environments.

## Introduction

Licorice (*Glycyrrhiza glabra* L.; family: Fabaceae) is an important, valuable plant which is globally known for its medicinal-industrial applicability. It contains various valuable metabolites such as triterpenoid saponins and flavonoids, especially glycyrrhizic acid (GA), glabridin, and rutin, which have numerous applications in the pharmaceutical, cosmetic, and food industries. Licorice extracts usually exhibit antimicrobial, anti-inflammatory, antispasmodic, anti-ulcer, expectorant, and antiviral effects which reflect their secondary metabolites^1^. Licorice is an endangered species for two reasons; human overharvesting from wild habitats and the prevalence of various types of abiotic stress. Licorice species are usually stress-tolerant, although progressive stress can ultimately lead to a decline in yield, with negative impacts on secondary metabolite production^2^. Salinity is a notable agricultural constraint that limits overall productivity and food security. It is estimated to affect approximately 50% of agricultural lands by 2050^3^. The salinization of agricultural land occurs mainly due to an accumulation of salts, especially sodium (Na^+^) and chloride (Cl^−^) ions in the soil^4^. A high level of Na^+^ accumulation is usually followed by limitations on water conductivity, soil porosity, and aeration, thereby restricting plant growth and causing an imbalance among ions, which is associated with higher levels of lipid peroxidation, more reactive oxygen species (ROS) and greater membrane permeability^2,5^. Plants can use several mechanisms to adapt to salinity and, thus, survive under salt stress. These functions operate in biochemical and physiological aspects^6^. Excessive NaCl uptake usually leads to osmotic stress, water deficit, and ionic imbalance, all of which have negative impacts on plant growth and survival^7^. In addition to plant susceptibility to salt stress, geographic origin and genetic foundations have reportedly influenced plant physiology and plant metabolites. Thus, selecting appropriate accessions from an available collection can be a valuable approach for target-based breeding and research^8^. A few licorice accessions were previously recommended for the remediation of abandoned salt-affected areas^9^. Another approach for mitigating the negative effects of stress on plants is the use of biological fertilizers which can be considered in programs for domestication, cultivation, and plant development. At the plant–microbe interface, the rhizosphere is a rich habitat of microorganisms that interact with plants, stimulate numerous signaling processes and contribute to material exchange^10^. Microorganisms and plants interact in a variety of ways that can result in neutral, synergistic, or undesirable consequences, with notable effects on the plants and the microbial population^11^. Plant growth-promoting bacteria (PGPB) are one of the important microorganisms that inhabit the rhizosphere in natural ecosystems. Their importance is partly due to their ability to promote plant growth by biologically-fixing nitrogen, enhancing soil nutrient content, producing siderophores and phytohormones, inducing systemic resistance, dissolving phosphates, and promoting symbioses between plants and microbes^11–14^.

While considering the above explanations, the present study was aimed at identifying different Iranian licorice accessions under integrated salt stress and *Azotobacter* sp. inoculation. Ultimately, they were examined for morphophysiological, biochemical, and nutritional properties. Generating such information can shed light on the provision of better opportunities for producing licorice roots under abiotic stress. Here, salinity was selected as an abiotic stress for its predominant occurrence in many parts of the world where licorice cultivation can be potentially productive.

## Materials and methods

### Experimental design and plant materials

**Figure 1:**
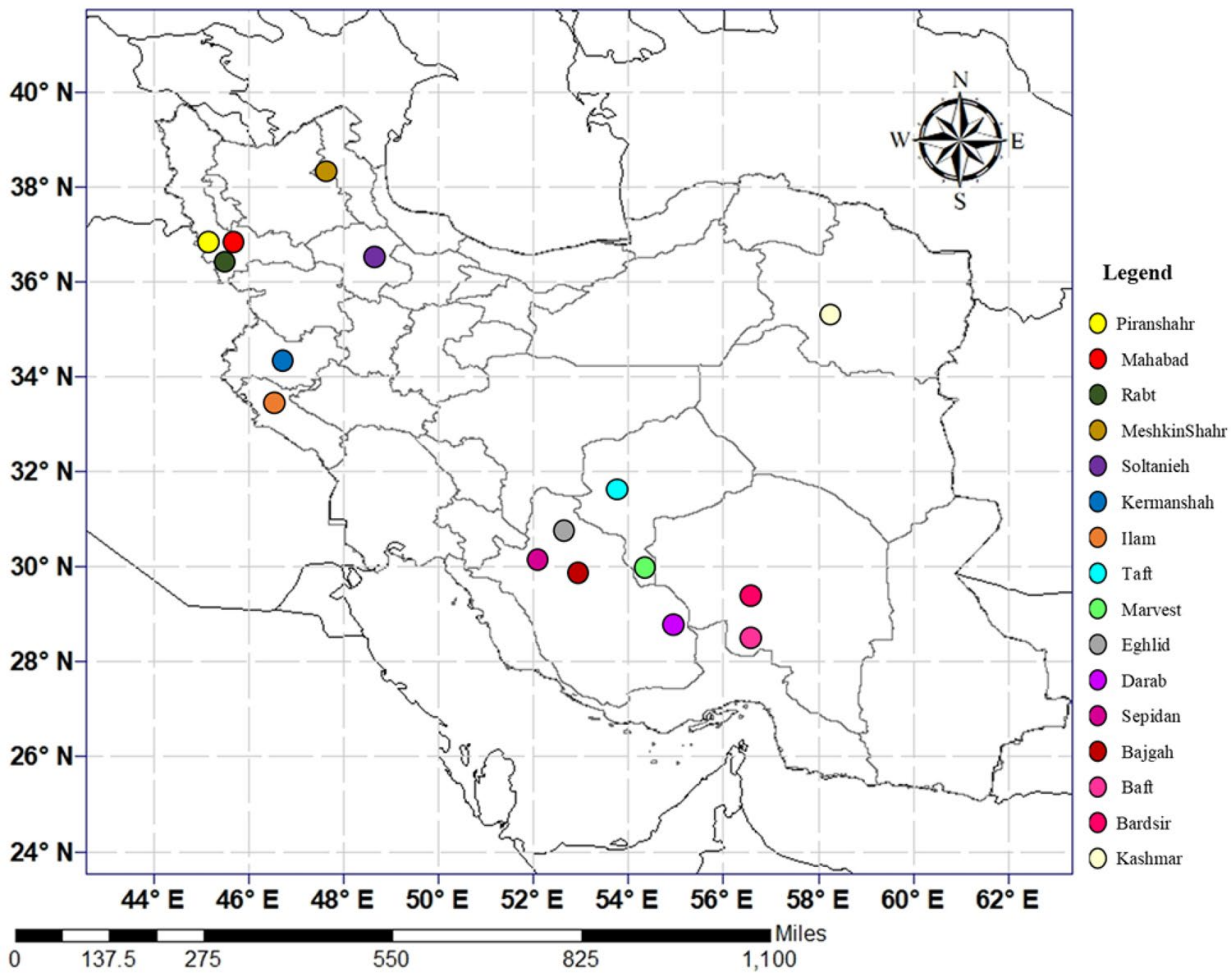
The collection areas for the investigated populations of *G. glabra* (the population's codes are visible in Table 1 and this figure is created using ArcGIS 10.8; URL: https://support.esri.com/en/products/desktop/arcgis-desktop/arcmap/10-8).

This study was conducted in a greenhouse of the School of Agriculture, Bajgah Region, Shiraz University, Shiraz, Iran, at a geographical location of 29°43′37.77" N and 52°35′12.84" E. The treatments included two levels of *Azotobacter* sp. (i.e., no-bacteria (control) and bacterial treatment), two levels of salinity (no-salt treatment (control) and salt treatment at 200 mM NaCl), and sixteen accessions of Iranian licorice plants (Table 1). Thus, sixty-four treatments with three replicates were applied, corresponding to a total of 192 pots. The rhizomes of sixteen licorice accessions were gathered from natural habitats where this plant existed wildly in 16 distinct regions of Iran (Table 1, Fig. 1). The collection of samples was carried out under national and scientific guidelines as described by Esmaeili et al. ^15^, and was based on International Standards for Sustainable Wild Collection of Medicinal and Aromatic Plants (ISSC-MAP) (Version 1.0), according to the Medicinal Plant Specialist Group Species Survival Commission of IUCN (The World Conservation Union). The permission to collect licorice plants from wild natural resources was obtained from the Iranian Organization of Forests and Rangeland (Government Organization). Upon collection, the accessions were conserved in an *ex-situ* conservation field in the School of Agriculture, Shiraz University, Shiraz, Iran (Longitude: 52°35′17.98″E, Latitude: 29˚43′26.14″N and Altitude: 1798 (m)). The specifics of maintenance were based on a scientific guideline as described by Esmaeili et al. ^15^. All plants are identified by a botanist (Dr. Ali Sonboli) from Shahid Beheshti University. The voucher specimens of the plants were deposited at the herbarium of the Medicinal Plants and Drugs Research Institute, Shahid Beheshti University, Evin, Tehran, Iran (Table1). Rhizome cuttings were made from the conserved plants and were cultivated in pots. The procedure of adaptation took place in a greenhouse (16 h of light, 25–28 °C, 120 µmol m^−2^ s^−1^ photon flux density, and 8 h of darkness, 18–21 °C). One year later, the adapted accessions were propagated using rhizome cuttings. Rhizomes were cut into equal-sized pieces (15 cm long and 2 cm in diameter) with sharp garden shears, and were dipped into a fungicide solution (Benomyl 1%). They were cultured in disinfected plastic pots (35 × 31 cm) with a prepared soil mixture (i.e., sieved field soil and sand at 1:1 ratio). Table S1 lists the physicochemical properties of the soil. After one year, the rooted cuttings of all accessions were selected for inclusion in the experiment.

**Table 1:** Origin, Geographical characteristics of different *G. glabra* accessions collected from Iran.

No	Accessions	Province	Longitude (E)	Latitude (N)	Altitude (m)	Voucher Number
1	Eghlid	Fars	52°29′37.9″	30°44′ 30.8″	2319	MPH-2670–1
2	Bajgah	Fars	52°35′ 17.98″	29°43′ 26.14″	1798	MPH-2670–2
3	Darab	Fars	54°25′ 37.64″	28°43′ 3.95″	1081	MPH-2670–3
4	Sepidan	Fars	52°00′ 41.5″	30°13′ 21.5″	2157	MPH-2670–4
5	Ilam	Ilam	46°17′ 43.72″	33°40′ 49.64″	1032	MPH-2670–5
6	Baft	Kerman	56°27′ 57.6″	29°15′ 7.1″	2241	MPH-2670–6
7	Bardsir	Kerman	56°15′ 21.94″	29°52′ 40.41″	2338	MPH-2670–7
8	Kashmar	Khorasan Razavi	58°27′ 51.07″	35°23′ 59.70″	1632	MPH-2670–8
9	Kermanshah	Kermanshah	46°59′ 21.37″	34°23′ 05.91″	1371	MPH-2670–9
10	MeshkinShahr	Ardabil	47°42′ 54.80″	38°25′ 01.10″	1412	MPH-2670–10
11	Taft	Yazd	53°50′ 59.3″	31°39′ 44.1″	2286	MPH-2670–11
12	Marvest	Yazd	54°13′ 51.9″	30°26′ 59.8″	1542	MPH-2670–12
13	Soltanieh	Zanjan	48°44′ 19.40″	36°24′ 40.21″	1842	MPH-2670–13
14	Rabt	West Azarbaijan	45°31′ 54.68″	36°12′ 41.35″	1075	MPH-2670–14
15	Piranshahr	West Azarbaijan	45°07′ 54.92″	36°37′ 42.95″	1492	MPH-2670–15
16	Mahabad	West Azarbaijan	45°43′ 09.53″	36°48′ 12.68″	1410	MPH-2670–16

### Bacterial solution preparation and treatments application

Bacteria from the genus *Azotobacter* sp. (endemic to Iran, from the Iranian Research Organization for Science and Technology (IROST), Persian Type Culture Collection (PTCC)) were cultured in a nutrition broth medium (NB) on a temperature-controlled shaker for 24 h at 28 ± 2 °C. At 600 nm, the bacterial density was measured. An inoculum of 106 CFU/mL was generated by centrifuging the freshly developed bacterial culture at 10,000 × rpm for 5 min for inoculation with *Azotobacter* sp. The resultant suspensions were used on licorice roots^13^. Specifically, the bacterial solution was sprayed on the roots of cuttings until the roots became completely wet. In no-bacterial treatment, the roots were sprayed with distilled water. They were then cultivated in pots (25 kg) and maintained for 11 months, before salinity treatment. The salt-treated plants were irrigated every five days with a salt solution of 200 mM. To prevent osmotic shock, the salt solution was added to the pots gradually, meaning that concentrations of 50, 100 and 150 mM were added to the pots before reaching the final concentration (200 mM). Plants of the control group and salt treatment were kept under saline stress in a greenhouse (16 h light, 25–28 °C, 120 µmol m^−2^ s^−1^ photon flux density, and 8 h of dark, ~ 15–21 °C for two months). Finally, the aerial parts and roots of the plants were harvested from all pots. Fresh leaves and roots were separated and stored at − 80 °C. After separating some leaves and roots from specific and similar parts of each plant, the separated samples were dried and stored. Dry and fresh weights of the roots were measured with a four-digit scale^13^.

### Glycyrrhizic acid and glabridin extraction and quantification

Dried roots (500 mg) were extracted using ethanol: water (70:30), followed by sonication for half an hour in an ultrasonic bath. The filtered extracts were centrifuged and analyzed by high-performance liquid chromatography (HPLC)^15^. The contents of glycyrrhizic acid and glabridin in the roots were determined using an Agilent liquid chromatography instrument, consisting of a 2695 separation module (Germany) equipped with a 100 μl loop and photodiode array detectors (PDA) using a C_18_ column (Knauer, 25 cm × 4.6 mm Eurospher 100–5) with an aqueous solution of 0.3% H_3_PO_4_ (solvent A) and with acetonitrile (solvent B) as the mobile phase. The flow rate was 1 mL/min with a linear solvent gradient of A–B as follows: 80% A for 10 min; reduced to 20% in 30 min and maintained for 5 min. The samples were measured at a wavelength of 230 nm for glabridin and 250 nm for glycyrrhizic acid. Each sample had a 20 μl injection volume for HPLC analysis. Millennium 32 (software) was used for data acquisition. The calibration curves for the standards were created by plotting the peak areas against the concentrations^15^.

### Rutin determination in aerial parts

Dried leaves (0.5 g) were extracted with ethyl alcohol (2 mL). After sonication (15 min) and centrifugation (10 min at 12,000 rpm), the extract was filtered and again dissolved in a hydroalcoholic solution (70:30). It was fractionated with petroleum ether (1 mL), ether (1 mL), ethyl acetate (1 mL), and n-butanol (1 mL). Rutin was quantitatively extracted in the ethyl acetate and n-butanol fractions, which were combined and dissolved in methanol (2 mL). The spectrophotometer was used for measuring the absorbance at 254 nm^17^.

### Determination of total phenols, flavonoids, and flavonols

Total phenolic content of ethanolic extracts was determined by a spectrophotometer and with a Folin-Ciocalteu reagent (Sigma-Aldrich, Darmstadt, Germany). Briefly, the extract (150 µl) was diluted with 2% Na_2_CO_3_ (1.5 mL) and was mixed for 3 min. Then, the Folin-Ciocalteu reagent was deposited in the tube and kept in darkness for 30 min. For the calibration curve, different concentrations of gallic acid were prepared as ethanolic solutions. The absorbance of the samples and the standard were measured at 750 nm via the spectrophotometer^18^. Regarding total flavonoid content, methanolic extracts were measured for absorbance at 510 nm. Catechin was used as a standard for the determination of total flavonoids^19^. Meanwhile, flavonols were measured by extracting dried crushed roots (0.5 g) with 3 mL ethyl alcohol (80%) (maceration, 24 h at room temperature). The suspension was filtered through a paper filter. The resultant solution (1 mL) was mixed with 1 mL aluminum chloride (2%) in ethanol (95%). After 20 min, the optical density of the solution was measured at 390 nm^20^.

### Total tannin and protein determination

The total tannin content of the ethanolic extract was measured by the Folin-Denis photometric assay. The extract (1 mL) was placed in a 100 mL volumetric flask. Folin–Denis reagent (5 mL) and sodium carbonate solution (10 mL) were added, and then diluted to a volume of 25 mL using distilled water. Finally, the solution was mixed well and incubated for 30 min at room temperature. The absorbance was measured at 760 nm. Total tannin content was measured using the standard tannic acid calibration curve^21^. Total protein was extracted from fresh leaves and the amount was determined by Bradford’s assay, followed by measurements of absorbance at 595 nm ^22^.

### Total sugar and starch

Quantification of total sugar content in the roots was performed according to a procedure by Zavřel et al.^22^. For total sugar extraction, 100 mg of dried-ground sample was poured into a microtube, and 2 mL ethanol (80%) was added. Then, the solution was maintained at room temperature for a night. Decanted into another 15-mL tube after centrifugation at 3000 rpm for 10 min, the residue was stored in the centrifuge tube. This extraction was repeated two more times, and then ethanol (80%) was added to the supernatant in a 15 mL volumetric flask. The total soluble sugar content of this extract was determined. The absorbance was measured at 490 nm^23^. For the measurement of starch, the residue in the test tube from total sugar extraction was used and the absorbance was read at 630 nm ^23^.

### Mineralogical analysis by ICP-OES and Kjeldahl

The Kjeldahl method was used for determining the nitrogen content in the leaves and roots. For total nitrogen, dry leaf biomass (0.5 g) was mixed with 1 g of a catalyst mixture (K_2_SO_4_, CuSO_4_.5H_2_O, and Se) and 10 mL sulfuric acid (98%) was used for digesting it. With distilled water, the volume was adjusted to 100 mL after mineralization. The distillate was made by transferring 40 mL of the solution to Kjeldahl flasks with a few drops of NaOH (8 N). Sulfuric acid (0.05 N) was used for titrating the distillate^24^.

Each dried powdered sample (500 mg) was placed in a PTFE digestion vessel containing 2 mL of modified aqua regia solution (HNO_3_ + HCL + H_2_O_2_; 1:3:1) which was kept for 10 min at room temperature. The sample was digested for 15 min in an ultrasonic bath digestion apparatus (model: 30 AL; frequency: 40 HTZ). Deionized water (25 mL) was added to the extract and centrifuged for 10 min at 12,000 rpm. The supernatant was removed for elemental analysis (Fe, P, K, Na) by ICP-OES ^25^.

The chlorine content in leaves and roots was determined according to a method used by Chapman and Pratt (1961). The chlorine content in each sample was calculated by the following formula: %Cl = (V AgNO_3_-VC) (%5 × 35.5 × 100/ WS). In the above formula, V AgNO_3_ is the volume of silver nitrate (mL) for titrating each sample. VC is the volume of silver nitrate (mL) used for the titration of the control sample, and WS is the weight of each sample (g)^26,27^.

### Statistical analysis

Minitab v. 18 software was used for statistical analysis. The experiments were conducted in a completely randomized design based on a factorial experiment with three replicates. The factors were licorice accessions, salinity, and bacterial inoculation. Analysis of variance (ANOVA) assisted in the analysis of experimental data, followed by Tukey’s multiple range test for mean comparisons at a confidence level of 95% (*p* ≤ 0.05). The slice approach was employed for mean comparisons where significant interactions were existent. Minitab software was used for principal component analysis based on a correlation matrix. Corrplot was created using R software version 4.1.1 (corrplot version 0.92^27^).

## Results

### Evaluation of root fresh and dry weight in different accessions of licorice under salt and Azotobacter treatments

**Figure 2:**
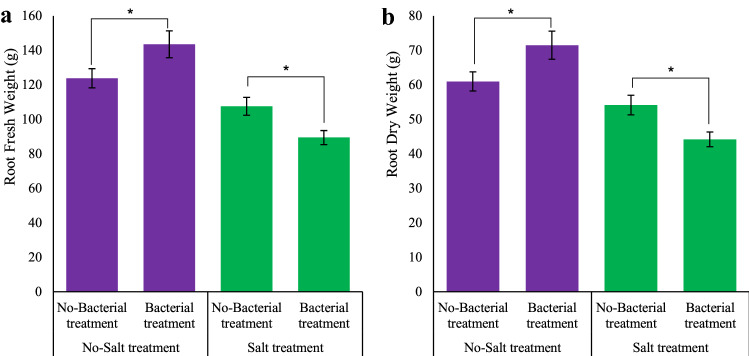
Measured parameters variation under studied treatments. (**a)** Root fresh weight, (**b)** Root dry weight. According to the analysis of variance, only multiple effects of *Azotobacter* and salinity showed a significant difference, just its mean comparison is shown. Mean values with the same letters are not significantly different (*p* ≤ 0.05), Tukey test. Bars stand for standard error (SE).

As shown in Figs. 2a, b, bacterial inoculation significantly (*p* ≤ 0.05) enhanced root fresh and dry biomass (with increases of 15.92 and 17.26%, respectively). Also, integrated bacterial inoculation in combination with salt stress significantly (*p* ≤ 0.05) increased these two parameters by 20.28 and 22.53%, respectively. Maximum root dry biomass (71.51 g) was achieved in response to the bacterial treatment without salinity (Fig. 2b).

### Secondary metabolites (Glycyrrhizic acid, glabridin, and rutin) in response to Azotobacter and salt stress

**Figure 3:**
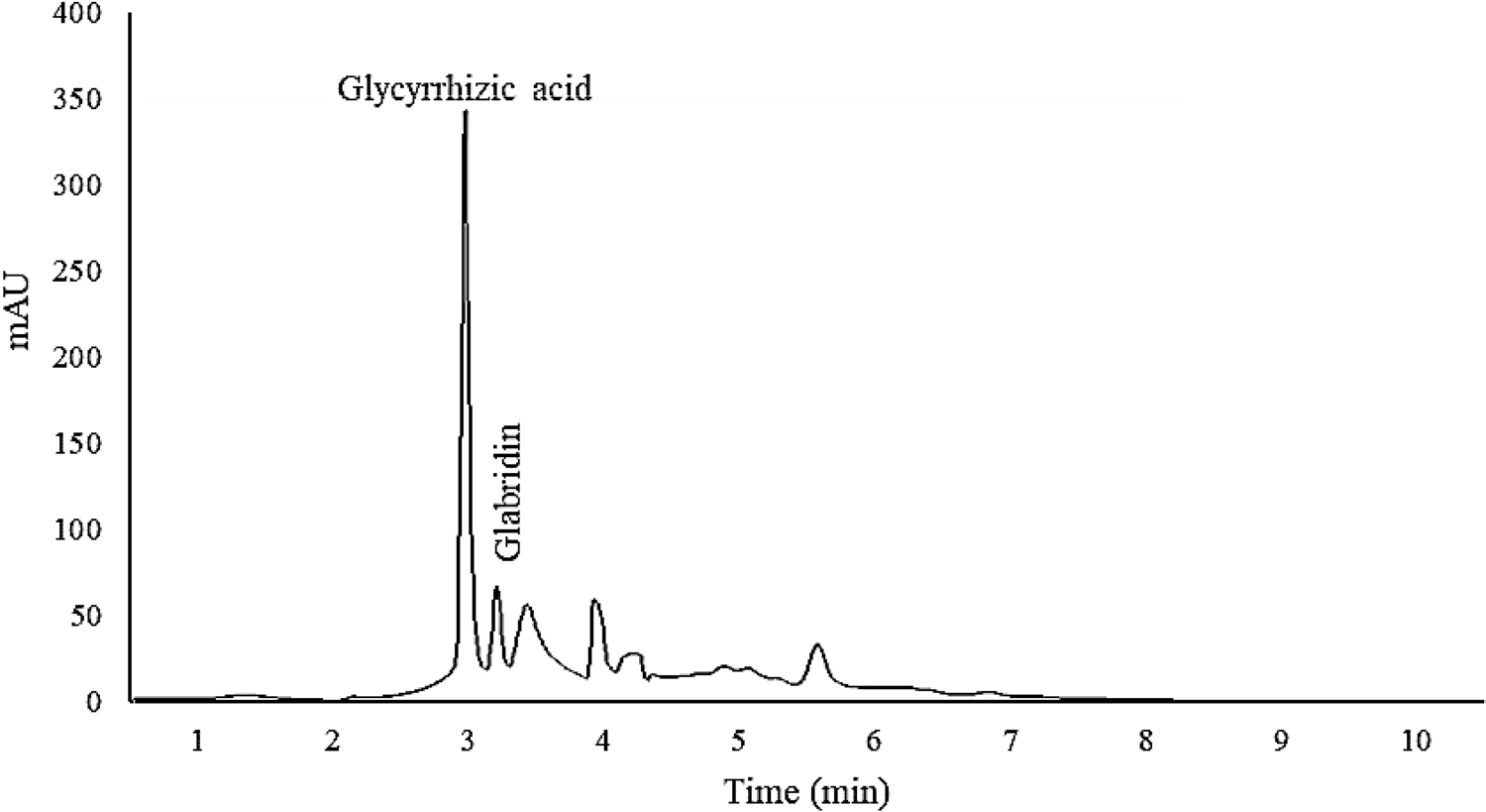
The HPLC chromatogram of Glycyrrhizic acid and Glabridin from one of the studied accessions (Baft accession).

**Figure 4:**
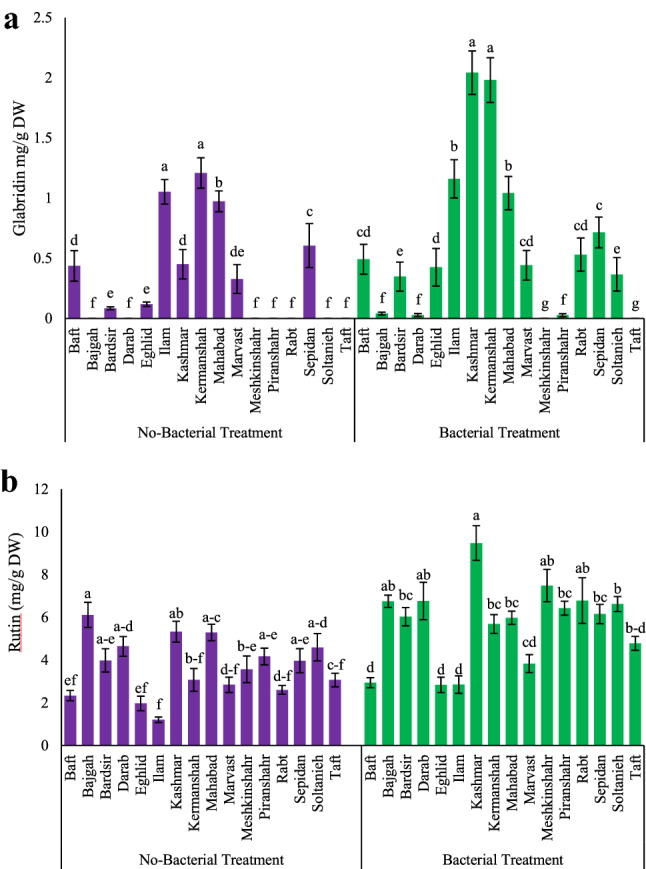
Measured parameters variation under studied treatments. (**a)** Glabridin, (**b)** Rutin. According to the analysis of variance, only multiple effects of *Azotobacter* and accessions showed a significant difference, just its mean comparison is shown. Mean values with the same letters are not significantly different (*p* ≤ 0.05), Tukey test. Bars stand for standard error (SE).

In the present study, GA was measured as the major secondary metabolite of licorice. Integrated bacterial inoculation and salt stress were able to significantly increase the GA content in the accessions. Among them, Bajgah and Sepidan accessions had the highest GA contents (96.26 and 83.17 mg/g DW, respectively), while the Eghlid accession had the lowest amount (41.98 mg/g DW). Under salt stress, the Baft accession showed maximum increase (173.53%) in GA by bacterial inoculation, compared to no bacterial inoculation (Fig. 3, Table 2). Another valuable metabolite in licorice, glabridin, was significantly (*p* ≤ 0.05) affected by bacterial application on the accessions (Fig. 3, 4a). The highest amount of glabridin among the accessions was observed in samples from Kermanshah and Ilam (16.42 and 10.85 mg/g DW, respectively). Under bacterial application, the maximum amount of glabridin was obtained in two accessions from Kashmar and Kermanshah (2.04 and 1.98 mg/g DW, respectively) (Fig. 4a). Observations on rutin content in the aerial parts of licorice showed that two accessions from Bajgah and Kashmar had higher amounts of rutin (6.11 and 9.48 mg/g DW, respectively), compared to the rest of the accessions (Fig. 4b).

**Table 2:** Effect of *Azotobacter* and salinity stress interactions on measured metabolites of 16 Iranian Licorice accessions.

Accessions	Traits											
	Flavonol (mg/g DW)				Phenol (mg/g DW)				Glycyrrhizic acid (mg/g DW)			
	No-Bacterial treatment		Bacterial treatment		No-Bacterial treatment		Bacterial treatment		No-Bacterial treatment		Bacterial treatment	
	No-Salt treatment	Salt treatment	No-Salt treatment	Salt treatment	No-Salt treatment	Salt treatment	No-Salt treatment	Salt treatment	No-Salt treatment	Salt treatment	No-Salt treatment	Salt treatment
Baft	0.171^a^ ± 0.033	0.205^a^ ± 0.036	0.225^a^ ± 0.012	0.443^a^ ± 0.048	1.46^b^ ± 0.15	3.18^a^ ± 0.19	2.61^ab^ ± 0.60	3.39^b-d^ ± 0.49	25.03^d^ ± 0.35	29.77f. ± 4.65	34.56^e^ ± 2.93	81.43f. ± 4.8
Bajgah	0.152^ab^ ± 0.027	0.161^ab^ ± 0.031	0.213^ab^ ± 0.036	0.168^b^ ± 0.036	1.89^ab^ ± 0.32	2.22^ab^ ± 0.61	3.22^ab^ ± 0.31	2.74^d^ ± 0.04	60.79^a^ ± 6.2	61.77^a-d^ ± 2.65	68.94^a-c^ ± 8.6	96.26^a^ ± 2.94
Bardsir	0.114^b^ ± 0.024	0.146^b^ ± 0.019	0.167^ab^ ± 0.018	0.266^b^ ± 0.034	1.89^ab^ ± 0.25	2.44^ab^ ± 0.28	2.67^ab^ ± 0.27	2.81^d^ ± 0.49	58.26^ab^ ± 8.7	70.81^a^ ± 13.95	75.25^a^ ± 9.15	56.52^ab^ ± 0.3
Darab	0.166^ab^ ± 0.026	0.173^ab^ ± 0.006	0.152^b^ ± 0.010	0.199^b^ ± 0.018	1.54^ab^ ± 0.09	2.18^ab^ ± 0.23	1.72^b^ ± 0.24	3.63^a-d^ ± 0.35	39.16^b-d^ ± 4.5	40.78^c-f^ ± 9.15	51.75^b-e^ ± 0.29	56.52^d-f^ ± 7.4
Eghlid	0.117^ab^ ± 0.01	0.151^ab^ ± 0.009	0.186^ab^ ± 0.019	0.181^b^ ± 0.016	2.54^a^ ± 0.13	2.54^ab^ ± 0.22	2.73^ab^ ± 0.43	3.85^a-d^ ± 0.82	31.29^d^ ± 0.28	36.97^ef^ ± 4.5	37.39^de^ ± 7.6	41.98f. ± 1.1
Ilam	0.132^ab^ ± 0.036	0.143^b^ ± 0.034	0.205^ab^ ± 0.028	0.164^b^ ± 0.019	1.57^ab^ ± 0.31	2.53^ab^ ± 0.20	2.31^ab^ ± 0.42	2.36^d^ ± 0.78	29.32^d^ ± 0.45	40.64^c-f^ ± 8.8	47.73^b-e^ ± 5.8	56.79^c-f^ ± 2.42
Kashmar	0.166^ab^ ± 0.024	0.179^ab^ ± 0.025	0.202^ab^ ± 0.013	0.209^b^ ± 0.027	1.52^ab^ ± 0.22	2.16^ab^ ± 0.67	2.90^ab^ ± 0.11	3.67^a-d^ ± 0.23	30.48^d^ ± 11	39.12^d-f^ ± 0.91	51.32^b-e^ ± 11.85	62.37^c-e^ ± 1.6
Kermanshah	0.153^ab^ ± 0.031	0.153^ab^ ± 0.018	0.213^ab^ ± 0.033	0.191^b^ ± 0.018	1.98^ab^ ± 0.52	2.07^ab^ ± 0.60	2.21^ab^ ± 0.55	4.31^a-d^ ± 0.25	19.75^d^ ± 2.5	23.49f. ± 1.7	40.84^de^ ± 4.83	44.78^ef^ ± 3.25
Mahabad	0.161^ab^ ± 0.016	0.177^ab^ ± 0.025	0.158^ab^ ± 0.034	0.188^b^ ± 0.024	1.73^ab^ ± 0.25	2.40^ab^ ± 0.10	2.78^ab^ ± 0.07	3.09^ cd^ ± 0.23	30.19^d^ ± 6.2	44.99^b-f^ ± 12	46.27^c-e^ ± 5.15	56.83^c-f^ ± 0.02
Marvast	0.126^ab^ ± 0.019	0.173^ab^ ± 0.03	0.180^ab^ ± 0.041	0.190^b^ ± 0.021	2.37^a^ ± 0.59	2.40^ab^ ± 0.65	4.05^a^ ± 0.42	3.99^a-d^ ± 0.37	54.29^a-c^ ± 3.55	55.78^a-e^ ± 1.4	58.18^a-d^ ± 3.66	59.25^c-f^ ± 1.29
Meshkinshahr	0.159^ab^ ± 0.022	0.160^ab^ ± 0.028	0.177^ab^ ± 0.013	0.194^b^ ± 0.006	1.47^ab^ ± 0.17	1.99^b^ ± 0.44	2.48^ab^ ± 0.33	3.07^ cd^ ± 0.08	37.32^b-d^ ± 6.75	40.38^c-f^ ± 12	41.04^de^ ± 0.57	60.81^c-f^ ± 8.5
Piranshahr	0.142^ab^ ± 0.007	0.155^ab^ ± 0.020	0.161^ab^ ± 0.001	0.174^b^ ± 0.034	1.59^ab^ ± 0.34	2.24^ab^ ± 0.67	2.51^ab^ ± 0.32	5.31^a-c^ ± 0.55	36.92^b-d^ ± 8.2	39.41^d-f^ ± 8	41.16^de^ ± 1.54	47.42^ef^ ± 1.82
Rabt	0.173^a^ ± 0.035	0.182^ab^ ± 0.044	0.209^ab^ ± 0.021	0.413^a^ ± 0.001	1.49^ab^ ± 0.2	2.67^ab^ ± 0.25	3.43^ab^ ± 0.56	5.48^ab^ ± 0.38	32.7^d^ ± 1	43.27^b-f^ ± 8.5	41.15^de^ ± 1.22	47.24^ef^ ± 6.25
Sepidan	0.145^ab^ ± 0.039	0.155^ab^ ± 0.017	0.169^ab^ ± 0.023	0.166^b^ ± 0.015	1.86^ab^ ± 0.2	2.18^ab^ ± 0.61	2.73^ab^ ± 0.94	3.30^b-d^ ± 0.24	55.6^ab^ ± 4.52	65.32^ab^ ± 8	76.7^a^ ± 8.65	83.17^ab^ ± 2.75
Soltanieh	0.124^ab^ ± 0.018	0.158^ab^ ± 0.03	0.167^ab^ ± 0.019	0.171^b^ ± 0.044	1.96^ab^ ± 0.09	2.53^ab^ ± 0.89	2.74^ab^ ± 0.69	5.90^a^ ± 0.23	39.02^b-d^ ± 2.3	70.31^a^ ± 7.5	69.47^ab^ ± 8.65	76.17^bc^ ± 0.7
Taft	0.147^ab^ ± 0.01	0.191^ab^ ± 0.010	0.160^ab^ ± 0.029	0.206^b^ ± 0.015	1.55^ab^ ± 0.31	3.07^a^ ± 0.48	3.40^ab^ ± 0.61	3.56^b-d^ ± 0.64	33.78^ cd^ ± 1.95	63.01^a-c^ ± 7.5	70.21^ab^ ± 9.25	72.67^b-d^ ± 0.35

According to the analysis of variance, the triple effects of *Azotobacter*, salinity, and accessions showed a significant difference, the slice method was used for mean comparisons. Mean values with the same letters within a column are not significantly different (*p* ≤ 0.05), Tukey test. Means ± standard error (SE).

### Integrated effect of Azotobacter and salt stress on total phenol, tannin, flavonoids, and flavonols

**Figure 5:**
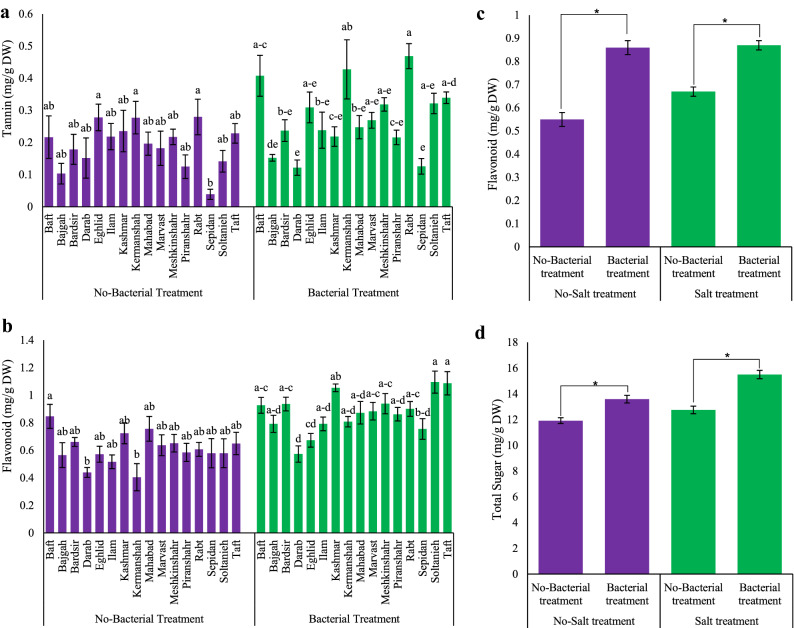
Measured parameters variation under studied treatments. (**a)** Tannin, (**b)** and (**c)** Flavonoid, and (**d)** Total sugar. According to the analysis of variance, only the multiple effects of *Azotobacter* and salinity showed a significant difference, just its mean comparison is shown. Mean values with the same letters are not significantly different (*p* ≤ 0.05), Tukey test. Bars stand for standard error (SE).

Phenolic content in licorice accessions varied depending on integrated bacterial inoculation and salt stress (Table 2). Regardless of bacterial inoculation, salinity significantly (*p* ≤ 0.05) enhanced phenolic content in all accessions (1.46–3.18 mg/g DW). Using bacteria on salt-treated plants increased total phenolic content in the Slotanieh accession (5.9 mg/g DW) (Table 2). Tannin content varied under bacterial inoculation among the accessions. The results revealed that the highest tannin content was observed in Kermanshah, Rabt, and Baft accessions (0.35, 0.32, and 0.29 mg/g DW, respectively), while the minimum quantity was observed in the Sepidan accession (0.082 mg/g DW) (Fig. 5a). According to the results (Fig. 5b), the highest percentage of flavonoids was observed in bacterial-inoculated plants of the Kashmar accession which contained 42% more flavonoids compared to the control treatment. Figure 5c shows that bacterial inoculation significantly increased flavonoid content under salt stress (*p* ≤ 0.05). An examination of flavonoid content showed that the highest increase (76%) was observed in the Rabt accession (Table 2).

### Total sugar examination under the combined effect of salt stress and Azotobacter inoculation

The lowest sugar content was observed in the control treatment (11.92 mg/g DW). Integrated salinity and bacterial application positively increased sugar content (15.5 mg/g DW) (Fig. 5d). In the absence and presence of salt stress treatments, bacterial inoculation enhanced total sugar content by 14.1 and 21.5%, respectively (Fig. 5d).

### Nutrient elements in Iranian licorice accessions under integrated Azotobacter and salt stress treatments

**Figure 6:**
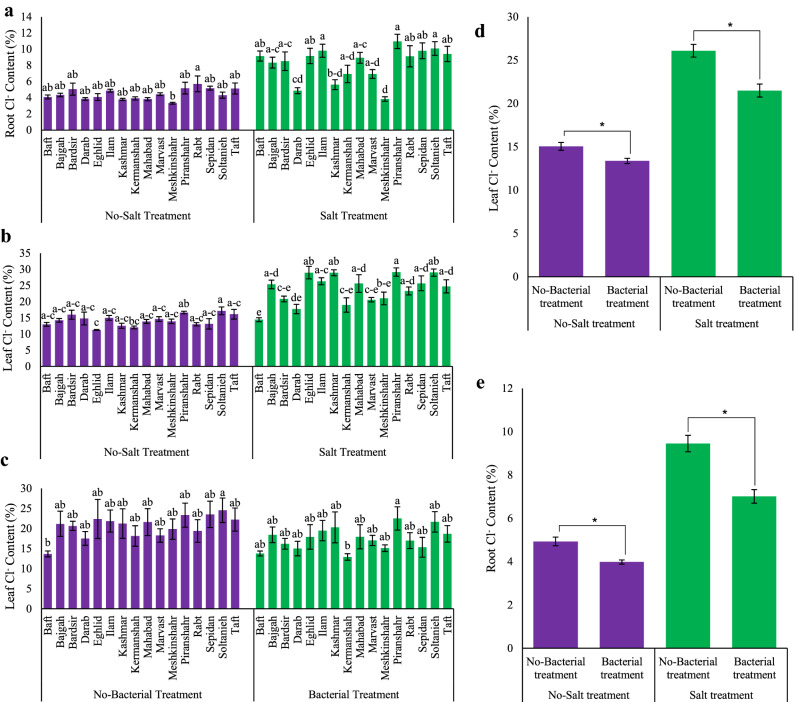
Measured parameters variation under studied treatments. (**a**, **e)** Root Cl^−^ content and (**b**, **c**, **d**) Leaf Cl^−^ content. According to the analysis of variance, only the multiple effects of *Azotobacter* and salinity showed a significant difference, just its mean comparison is shown. Mean values with the same letters are not significantly different (*p* ≤ 0.05), Tukey test. Bars stand for standard error (SE).

The results showed that N, P, K and Fe concentrations in licorice leaves decreased significantly under the effect of salt stress (*p* ≤ 0.05), whereas bacterial inoculation positively affected the concentrations of these elements. Regardless of salinity, the highest amount of each element was found in a different accession. Specifically, the highest amounts of leaf N (Piranshahr (3.61%)), P (Darab (70 ppm)), K (Sepidan (323 ppm)), and Fe (Darab and Meshkinshahr (8.5 ppm)) were detected under bacterial inoculation (Table S2, S3). An analysis of root elements after bacterial inoculation showed that Eghlid had the maximum Fe (6.7 ppm), Bardsir had the maximum K (137 ppm), and Darab had the maximum P (62 ppm) concentration (Table S4). Also, there were variations in Na^+^ and Cl^−^ among the accessions under the effect of salinity. The results showed that Na^+^ increased in both roots and leaves of licorice under salinity (Table S3), whereas bacterial inoculation attenuated Na^+^ and Cl^−^ contents. Under bacterial inoculation, there was a significant attenuation (*p* ≤ 0.05) of Na^+^ concentration in plant tissues of Rabt and Bardsir (with decreases of 59.01% and 50.79%, respectively) (Table S3). By salt stress, maximum Na^+^ content in licorice leaf was observed in Rabt (566% increase), while Meshkinshahr showed minimal Na^+^ accumulation (102.7% increase), compared to the control. Cl^−^ variations under salt stress showed that the leaves and roots had minimal Cl^−^ concentrations in Baft and Meshkinshahr accessions (Figs. 6a, b). Regardless of the salt stress, bacterial inoculation had a positive effect on the Cl^−^ concentration of all accessions. The minimum Cl^−^ content was observed in Meshkinshahr (Fig. 6c). Integrated bacterial inoculation and salinity also affected Cl^−^ content in both leaf and root. Thus, using bacteria did not significantly reduce Cl^−^ content, whereas bacterial inoculation reduced the Cl^−^ content in non-salt-treated licorice plants, both in the root and leaf (*p* ≤ 0.05) (Figs. 6d, e).

### Principal component and corrplot analyses of licorice accessions under the treatments

**Figure 7:**
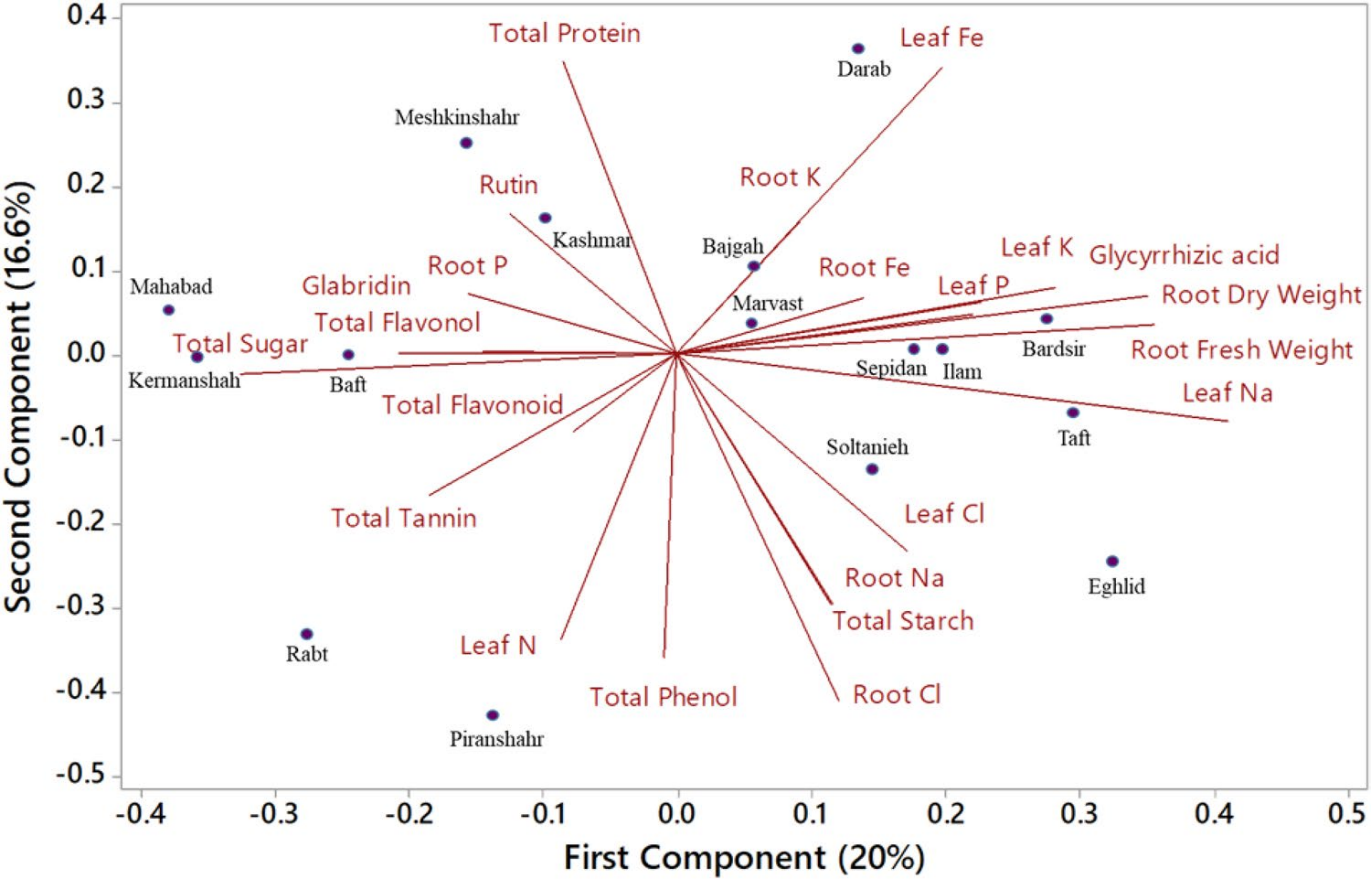
Principal component analysis of studied treatments on morphophysiological and biochemical characteristics of 16 Iranian licorice accessions.

In the biplot of PC analysis, the first two PCs represented 36.6% of the variation in the measured traits among treatments and accessions (Fig. 7, Table S5). The first PC explained 20% of the variation and included GA, fresh and dry root biomass, as well as leaf and root nutritional components. In contrast, the second PC explained 16.6% of the variation in traits and treatments, thereby comprising rutin, flavonoid, glabridin, and total sugars. The acute angles (< 90°) represent positive correlations, while negative correlations were represented by broad obtuse angles (90° <). In the licorice accessions, relations among root biomass, elemental analysis, and secondary metabolite production were evaluated using correlation analysis (Fig. S1). Stronger positive correlations were found between root fresh weight and dry weight. Among the parameters of secondary metabolites, total tannin and GA showed a significant negative correlation (Fig. S1).

## Discussion

Soil salinization is a serious concern that impacts thousands of hectares around the world. Microorganisms are capable of coping with salt stress and reducing its negative effects on plant growth^28^. PGPB has a tremendous ability to reduce salinity and enhance plant growth, which plays an important role in food security by increasing crop productivity^29^. Genotype selection is also a notable method for efforts to overcome salinity in cultivated areas^30^.

In the present study, biomass analysis revealed that bacterial inoculation significantly increased FW and DW of licorice roots (*p* ≤ 0.05). In line with the present results, the improvement in biomass under bacterial inoculation was also reported in previous studies on soybean (*Glycine max* L. Merrill)^31^ and white clover (*Trifolium repens* L.)^32^. PGPB directly assists in plant development by increasing nutrient absorption, creating phytohormones, or reducing plant ethylene levels enzymatically. One of the key processes by which bacteria affect plant growth is the production of auxins by root-associated microbes^33^. As observed in the current research, licorice biomass significantly (*p* ≤ 0.05) decreased under salinity stress, which confirms similar indications on *Tetraena mandavillei* L.^34^ and basil (*Ocimum basilicum* L.)^35^ in previous studies. This restriction in plant growth occurs through a reduction in the plant’s ability to absorb water, through osmotic stress, stunted growth, leaf burn, and wilting, which can possibly lead to death^36^.

Plant growth is aided by several mechanisms, i.e. PGPB through ACC deaminase activity, the manufacture of plant hormones such as IAA, gibberellic acid, abscisic acid, cytokinin, exopolysaccharides, and by the utilization of exopolysaccharides^37^.

Large differences between tolerant and sensitive plants could be attributed to signal reception and processing, as a result of genetic variability in complex traits. Various metabolic alterations occur in plants to help them adapt to the saline environment^38^. The results of the current study showed that higher amounts of GA were observed in two accessions (Bajgah and Bardsir). A previous study on *G. glabra* accessions led to similar results in some respects and showed genotype variations in root GA concentration^15,39^. Licorice has a major triterpenoid saponin, GA, which reportedly has antioxidant activity and plays a critical function in ROS scavenging, leading to a significant reduction in oxidative damage^40^. During salt stress, enhanced levels of GA in plants could be due to a reduction in root biomass^41^. Also, biosynthetic gene expression increased in bacterial-inoculated plants under salt stress, suggesting that the upregulation of GA biosynthetic genes was responsible for the enhancement in GA concentrations in stressed plants^42^. In agreement with the present results, GA levels changed remarkably depending on genotype, stress conditions, and bacterial inoculation in previous studies on *G. uralensis*^43^ and *G. glabra*^44,45^. Changes in licorice root composition may also be influenced by soil characteristics and by the growing environment. While this variation may have a genetic basis, the validation of this assertion requires that several populations of G. *glabra* be grown in the same habitat to exclude environmental impacts^13^. Flavonoids are a type of chemical defense that occur broadly in response to abiotic stresses. They exhibit a strong free radical scavenging function that contributes to the attenuation of oxidative stress in various crops^46^. Flavonoid metabolite synthesis has helped many plant species adapt to a variety of undesirable conditions^47^. A study on *Aegilops cylindrica* revealed that flavonoids can significantly improve adaptation to salt tolerance^48^. In the present study, variations in total flavonoid content were observed among licorice accessions. Specifically, integrated bacterial inoculation had a remarkable effect on increasing the amount of flavonoids in the roots of each licorice accession. As observed in the present study, the total flavonoid content increased significantly by bacterial inoculation under salt stress. By altering plant secondary metabolism, bacterial inoculation can develop plant resistance to abiotic stress. Consequently, bacterial inoculation can enhance the biosynthesis of health-promoting phytochemicals such as polyphenols and flavonoids^49^. Researchers found that bacterial-inoculated *G. uralensis*^50^ and *G. max*^51^ produced greater amounts of flavonoids and phenolics than non-inoculated plants. Similarly, bacterial inoculation under salinity was effective in increasing total phenolic content in the accessions of the present study. One of the most fundamental mechanisms that affect phytochemical production is the establishment of symbiosis between plant roots and helpful soil-borne bacteria that are ecologically and commercially important^52^. Numerous cases of research have shown that bacterial inoculation can affect the production of secondary metabolites in plants by altering terpenoid metabolism and the shikimate pathway, thereby increasing the biosynthesis of isoprenoids, polyketides, and polyphenols^51,53,54^. Also, bacterial inoculation reportedly increased the concentration of polyphenols and carotenoids, as well as the activity of antioxidant enzymes, mevalonate, malonyl-CoA, and shikimate pathways, thereby promoting the production of isoprenoids, polyketides, and polyphenols^55^. Stress tolerance is enhanced by such substances with significant antioxidant potential^56^. Tolerant plants can resist salinity by regulating inorganic ion quantity, and by translocating excess Na^+^ to aerial parts of the plant. Some accessions modify their ability to absorb water under the salt influence by decreasing water content in seedlings. This function may be regarded as an adaptive mechanism. Differences in the accumulation of polyphenols and other metabolites can be affected by environmental and genetic factors, as well as by extraction methods and postharvest procedures^57^.

Mitigation strategies that rely on bacterial inoculation can help alleviate salt stress in plants. As observed in this study, plant tissue N, P, K, and Fe concentrations decreased in almost all accessions under salinity, but increased by the effect of bacterial inoculation. A similar trend was previously observed in *Z. mays* L.^58^ and *Vicia villosa*^59^. High concentrations of salt inhibit bacterial colonization and cause root hair curling, infection filament formation, reduction in nodule respiration, and cytosolic leghemoglobin protein production. A low level of nodulation can affect N uptake negatively^60^. Research has shown that inoculation with rhizobia improves nodulation and nitrogen fixation by legumes under stress conditions. A higher level of nutrient uptake, caused by bacterial symbiosis, positively affected the nitrogenase enzyme function, resulting in enhanced nitrogen fixation and better plant growth^61^. Inoculation with beneficial bacteria boosts nutrient uptake by plants at times of salt stress, resulting in enhanced levels of chlorophyll pigment synthesis in the inoculated plants^62^. This statement is in agreement with the outcomes of the present research, in which bacterial inoculation significantly decreased N uptake. Salt-tolerant rhizobacteria can increase the uptake of K ions by inducing the expression of a high-affinity ion transporter (AtHKT1) in plants under salinity, resulting in a higher K^+^/Na^+^ ratio that promotes salt tolerance. Strains of PGPB directly modify plant growth by solubilizing P and K, while increasing nutrient uptake^63^. Also, there are indications that salt-tolerant *Azospirillum* restricts the Na^+^ influx into the roots and causes a high K^+^/Na^+^ ratio in salt-stressed plants. K plays a critical function in the osmotic adjustment of plants. Salinity leads to nutrient imbalance due to higher concentrations of Na^+^/Ca^2+^, Na^+^/K^+^, Na^+^/Mg^2+^, Cl^−^/NO_3_, and Cl^−^/H_2_PO_4_^−^, thereby retarding plant growth^64^. The irrigation of plants with saline water usually reduces infiltration, resulting in low uptake levels of plant-available water, thereby affecting plant performance and survival. It is also known that salt stress reduces Fe availability for plants, as observed in the current study. Similar results have also been stated in previous reports^65,66^. Since Fe can increase the uptake of other elements by roots and aerial parts under saline conditions, bacterial inoculation increased the Fe concentration in the leaves and roots of licorice. Thus, bacterial inoculation was able to increase the uptake and concentration of other elements including N, P, and K, thereby mitigating the effects of salt stress. The production of siderophores, as low molecular weight molecules, usually drives Fe absorption in microbial systems. Under extreme saline conditions, exopolysaccharides are a common PGPB metabolite. Extracellular polymeric substances (EPS) usually aid in microbial adhesion to plant roots, while assisting in biofilm formation to preserve cells from desiccation, as well as increasing the mobility of root-associated bacteria^67^. EPS can reduce ion toxicity in plants by minimizing NaCl influx through the expression of the HKT1/KC transporter. By affecting important metabolic processes in plants and maintaining soil physicochemical properties, EPS-producing bacteria may also contribute to improvements in plant productivity under saline conditions. The current results showed that bacterial inoculation was able to reduce Na^+^ and Cl^−^ concentrations in the shoots and roots of some licorice accessions through EPS production and ion uptake, as well as proline and polyphenol synthesis^51^. Growth enhancement in inoculated plants could be explained by a halt in Na^+^ transport. EPS generated by PGPB helped plants cope with salt stress. Thus, there is scope for their application as bio inoculants to boost rhizosphere colonization, soil quality, and nutrient absorption in saline soils. Also, EPS could be utilized as an additive in bio inoculants to protect PGPB from the initial stress caused by salinity^68^.

## Conclusion

The present findings showed variations in growth traits and secondary metabolites of *G. glabra* accessions because of cultivation under salinity stress and bacterial inoculation. *G. glabra* would be classified as a halophyte, based on our findings. Depending on the metabolites needed for food, pharmaceutical, or cosmetic industries, different populations of licorice can be used and grown with specific considerations, as mentioned in the present study. We concluded that Bajgah and Sepidan accessions had higher amounts of GA. Meanwhile, Kashmar and Kermanshah accessions had higher amounts of glabridin. Bajgah and Kashmar accessions had higher amounts of rutin under integrated bacterial inoculation and salt stress. In conclusion, a selection-based approach to genetic variation can associate positively with integrated biofertilizers and would be a helpful tool for the cultivation of licorice in saline soils.

## Supplementary Information

Supplementary Information.

## Data Availability

The dataset generated during and/or analyzed during the current study are available from the corresponding author on reasonable request.
