# Supplementary material for: Evaluation of metabolites in Iranian Licorice accessions under salinity stress and Azotobacter sp. inoculation
Source: Sci Rep. 2022 Sep 23;12:15837. doi: 10.1038/s41598-022-20366-6 (PMC9508240; doi:10.1038/s41598-022-20366-6)
Supplement: Supplementary file 1 — Supplementary Information. [file 41598_2022_20366_MOESM1_ESM.pdf]

Table S1. Physiochemical properties of the soil used in the present experiment.

Soil texture	Sand	Silt	Clay	OM*	N	Cu-DTPA	Mn-DTPA	Zn-DTPA	P-Olsen	K	Fe	EC	pH
Sandy-Clay	%					mg/kg						ds/m	-
	50	11	39	0.9	0.14	0.93	4.3	0.23	8	53	4.62	1.4	7.6

In the table *OM stand for organic matter.

Table S2. Effect of Azotobacter and salinity stress interaction on measured metabolites of 16 Iranian licorice accessions.

No.	Accessions	Leaf Nitrogen (%)				Leaf Potassium (ppm)				Leaf Phosphorus (ppm)			
		No-Bacterial treatment		Bacterial treatment		No-Bacterial treatment		Bacterial treatment		No-Bacterial treatment		Bacterial treatment	
		No-Salt treatment	Salt treatment	No-Salt treatment	Salt treatment	No-Salt treatment	Salt treatment	No-Salt treatment	Salt treatment	No-Salt treatment	Salt treatment	No-Salt treatment	Salt treatment
1	Baft	1.51^{h}±0.051	1.26^{fg}±0.052	1.79^{e-g}±0.051	1.32^{gh}±0.051	172^{f}±1.16	152^{c}±1	173^{g}±0.58	155^{e}±0.88	42^{bc}±0.52	27^{b-d}±1	49^{b}±0.52	33^{bc}±1
2	Bajgah	1.65^{f-h}±0.053	1.48^{d-f}±0.051	1.71^{fg}±0.057	1.60^{d-f}±0.057	203^{c}±0.52	153^{c}±0.57	217^{c}±1.1	172^{c}±1.1	42^{bc}±1.19	27^{b-d}±0.48	48^{b}±0.58	39^{b}±0.58
3	Bardsir	1.82^{e-g}±0.051	1.46^{d-g}±0.051	3.19^{ab}±0.052	1.54^{d-g}±0.051	175^{f}±1.15	163^{b}±0.44	269^{b}±1.82	172^{c}±1.4	26^{fg}±0.5	12^{i}±0.42	31^{e}±0.99	25^{d}±1
4	Darab	1.43^{h}±0.064	1.37^{e-g}±0.05	1.48^{g}±0.051	1.37^{f-h}±0.052	149^{h}±0.48	122^{f}±1.02	162^{h}±0.66	142^{g}±0.56	38^{cd}±1.16	31^{b}±0.52	70^{a}±1.1	37^{bc}±0.54
5	Eghlid	1.88^{d-g}±0.052	1.57^{de}±0.052	1.74^{fg}±0.032	1.76^{c-e}±0.05	186^{e}±1	128^{e}±0.48	210^{d}±1.12	142^{g}±1.18	57^{a}±0.48	43^{a}±1.1	69^{a}±1.64	50^{a}±1.19
6	Ilam	2.05^{b-e}±0.019	1.54^{de}±0.051	2.07^{d-f}±0.051	1.68^{c-e}±0.051	241^{b}±1.4	125^{ef}±0.5	268^{b}±1.88	180^{b}±0.58	22^{h}±0.48	19^{fg}±0.98	24^{f}±1	20^{ef}±0.42
7	Kashmar	1.40^{h}±0.057	1.20^{g}±0.057	1.68^{fg}±0.051	1.23^{h}±0.051	141^{i}±0.54	121^{f}±0.54	147^{i}±0.46	133^{h}±0.54	39^{c}±1	15^{h}±0.5	41^{c}±1	31^{c}±0.56
8	Kermanshah	1.91^{c-f}±0.047	1.46^{d-g}±0.064	1.99^{ef}±0.074	1.57^{d-g}±0.052	163^{g}±0.48	139^{d}±0.61	179^{f}±0.68	149^{f}±0.5	23^{gh}±1.14	20^{e-g}±1.12	30^{e}±0.58	23^{de}±0.52
9	Mahabad	2.30^{b}±0.057	1.93^{bc}±0.052	2.55^{cd}±0.052	2.16^{b}±0.051	92^{m}±0.42	85^{i}±0.46	114^{k}±0.44	92^{j}±0.4	23^{gh}±0.54	19^{fg}±0.54	24^{f}±0.58	20^{ef}±1.12
10	Marvast	1.62^{gh}±0.051	1.43^{e-g}±0.049	1.69^{fg}±0.057	1.51^{e-g}±0.051	186^{d}±1	173^{a}±1	206^{de}±1.19	197^{b}±0.58	29^{ef}±1	22^{d-f}±0.58	30^{e}±1.18	23^{de}±0.5
11	Meshkinshahr	1.93^{c-e}±0.006	1.68^{cd}±0.05	2.13^{d-f}±0.006	1.79^{cd}±0.057	110^{l}±0.56	91^{h}±0.44	144^{i}±0.5	59^{l}±0.56	23^{gh}±0.5	18^{f-h}±0.44	32^{e}±1.16	22^{d-f}±0.47
12	Piranshahr	3.25^{a}±0.051	2.69^{a}±0.006	3.61^{a}±0.05	2.70^{a}±0.005	81^{n}±0.38	55^{j}±0.34	96^{l}±0.48	73^{k}±1	25^{f-h}±0.5	17^{gh}±1	30^{e}±0.56	19^{f}±1
13	Rabt	2.16^{bc}±0.05	2.02^{b}±0.007	2.27^{de}±0.052	2.10^{b}±0.055	123^{k}±1.15	108^{g}±0.52	134^{j}±1.15	122^{i}±0.44	33^{de}±0.53	21^{e-g}±1.12	34^{de}±0.56	24^{de}±1.1
14	Sepidan	1.96^{c-e}±0.052	1.90^{bc}±0.059	1.99^{ef}±0.064	1.93^{bc}±0.051	258^{a}±1.62	165^{b}±0.58	323^{a}±1.28	224^{a}±1.22	46^{b}±0.52	24^{c-e}±0.48	49^{b}±1.18	31^{c}±0.54
15	Soltanieh	2.13^{b-d}±0.051	1.91^{bc}±0.054	2.83^{bc}±0.051	2.07^{b}±0.051	130^{j}±0.52	82^{i}±0.48	143^{i}±0.58	121^{i}±0.36	32^{e}±0.5	21^{ef}±0.54	37^{cd}±1.12	31^{c}±1
16	Taft	1.79^{e-g}±0.054	1.37^{e-g}±0.052	1.82^{e-g}±0.051	1.62^{d-f}±0.051	175^{f}±0.54	163^{b}±0.06	203^{e}±1	165^{d}±1	38^{cd}±1.18	29^{bc}±0.58	47^{b}±1.18	33^{bc}±0.52

According to the analysis of variance that triple effects of Azotobacter, salinity and accessions showed significant difference, slice method used for mean comparisons. Mean values with the same letters within a column are not significantly different ($p<0.05$), Tukey test. Means ± standard error (SE).

Table S3. Effect of Azotobacter and salinity stress interaction on measured metabolites of 16 Iranian licorice accessions.

		Traits											
		Root Sodium (ppm)				Leaf Sodium (ppm)				Leaf Iron (ppm)			
		No-Bacterial treatment		Bacterial treatment		No-Bacterial treatment		Bacterial treatment		No-Bacterial treatment		Bacterial treatment	
No.	Accessions	No-Salt treatment	Salt treatment	No-Salt treatment	Salt treatment	No-Salt treatment	Salt treatment	No-Salt treatment	Salt treatment	No-Salt treatment	Salt treatment	No-Salt treatment	Salt treatment
1	Baft	$12^{bc}\pm0.42$	$55^{c}\pm1.1$	$9.2^{bc}\pm0.11$	$35^{c}\pm1.14$	$24^{e-g}\pm0.84$	$84^{i}\pm0.65$	$23^{e-g}\pm0.8$	$53^{h}\pm0.82$	$3.8^{a-c}\pm0.019$	$1.8^{c-e}\pm0.002$	$4.1^{d}\pm0.109$	$3.4^{a-c}\pm0.112$
2	Bajgah	$5.8^{g}\pm0.06$	$32^{gh}\pm1.23$	$4.8^{h}\pm0.05$	$30^{de}\pm1.02$	$31^{d}\pm1$	$89^{hi}\pm0.92$	$29^{cd}\pm0.56$	$83^{f}\pm0.52$	$2.9^{ef}\pm0.011$	$1.8^{c-e}\pm0.006$	$3.5^{e}\pm0.107$	$2.7^{df}\pm0.014$
3	Bardsir	$9.5^{c-e}\pm0.11$	$63^{b}\pm1.18$	$7.4^{d-g}\pm0.07$	$31^{c-e}\pm0.98$	$49^{a}\pm1.22$	$107^{de}\pm1$	$44^{a}\pm1.2$	$66^{g}\pm0.11$	$3.7^{a-d}\pm0.119$	$1.6^{d-f}\pm0.002$	$5.5^{b}\pm0.112$	$3.6^{a-c}\pm0.132$
4	Darab	$6.6^{fg}\pm0.04$	$29^{hi}\pm0.88$	$6^{gh}\pm0.08$	$21^{g}\pm1$	$27^{d-f}\pm0.68$	$104^{ef}\pm1.2$	$25^{c-f}\pm0.9$	$90^{de}\pm0.98$	$4.2^{ab}\pm0.164$	$1.4^{e-g}\pm0.009$	$8.5^{a}\pm0.211$	$3.7^{ab}\pm0.118$
5	Eghlid	$11^{b-d}\pm0.21$	$76^{a}\pm2.26$	$8.9^{b-e}\pm0.1$	$63^{a}\pm2.44$	$32^{cd}\pm0.72$	$137^{a}\pm1.52$	$28^{c-e}\pm1$	$110^{b}\pm0.88$	$3.1^{de}\pm0.122$	$2.3^{bc}\pm0.111$	$3.5^{e}\pm0.102$	$2.9^{c-e}\pm0.098$
6	Ilam	$9.5^{c-e}\pm0.98$	$34^{g}\pm1.02$	$7.3^{e-g}\pm0.06$	$31^{c-e}\pm0.88$	$28^{de}\pm0.48$	$134^{a}\pm1$	$21^{f-h}\pm0.52$	$92^{cd}\pm0.18$	$3.5^{b-e}\pm0.118$	$2.6^{ab}\pm0.102$	$4.4^{cd}\pm0.173$	$2.7^{d-f}\pm0.066$
7	Kashmar	$8.5^{d-f}\pm0.12$	$28^{hi}\pm0.68$	$6.8^{fg}\pm0.03$	$27^{ef}\pm1.15$	$22^{f-h}\pm0.44$	$95^{g}\pm0.56$	$17^{hi}\pm0.64$	$92^{cd}\pm0.14$	$4.3^{a}\pm0.113$	$2.2^{bc}\pm0.106$	$5.1^{bc}\pm0.119$	$3.1^{b-d}\pm0.116$
8	Kermanshah	$11^{b-d}\pm0.16$	$49^{de}\pm1.16$	$8.3^{b-f}\pm0.18$	$28^{ef}\pm0.57$	$29^{de}\pm0.98$	$85^{hi}\pm0.44$	$21^{f-h}\pm0.6$	$56^{h}\pm0.56$	$2.2^{gh}\pm0.058$	$1.8^{c-e}\pm0.002$	$2.6^{f}\pm0.045$	$1.9^{gh}\pm0.092$
9	Mahabad	$9.3^{de}\pm0.18$	$52^{cd}\pm2$	$7.6^{c-g}\pm0.15$	$33^{cd}\pm1.18$	$22^{f-h}\pm0.54$	$66^{k}\pm0.9$	$18^{g-i}\pm0.44$	$56^{h}\pm0.34$	$4^{ab}\pm0.146$	$1.8^{c-e}\pm0.088$	$4.1^{d}\pm0.124$	$3.1^{b-d}\pm0.119$
10	Marvast	$13^{ab}\pm0.22$	$25^{i}\pm0.96$	$11^{a}\pm1.16$	$22^{g}\pm0.77$	$41^{b}\pm1.2$	$101^{f}\pm1.2$	$35^{b}\pm1.12$	$87^{d-f}\pm0.77$	$2.3^{gh}\pm0.108$	$2.2^{bc}\pm0.41$	$4.2^{d}\pm0.118$	$2.2^{fg}\pm0.1$
11	Meshkinshahr	$8.1^{e-g}\pm0.08$	$42^{f}\pm1.15$	$7.2^{e-g}\pm0.05$	$27^{ef}\pm1.15$	$37^{bc}\pm1$	$75^{j}\pm0.64$	$30^{bc}\pm0.32$	$40^{i}\pm1.16$	$3.7^{a-d}\pm0.116$	$2.4^{ab}\pm0.101$	$8.5^{a}\pm0.223$	$3.2^{b-d}\pm0.064$
12	Piranshahr	$8.3^{e-g}\pm0.04$	$46^{ef}\pm0.58$	$6.2^{gh}\pm0.07$	$35^{c}\pm1.64$	$19^{gh}\pm0.88$	$90^{gh}\pm1$	$17^{hi}\pm0.09$	$65^{g}\pm0.48$	$2.5^{fg}\pm0.052$	$1.1^{g}\pm0.1$	$2.7^{f}\pm0.114$	$2.4^{ef}\pm0.141$
13	Rabt	$9.7^{c-e}\pm0.1$	$61^{b}\pm1.08$	$9.6^{ab}\pm0.1$	$25^{fg}\pm1.16$	$18^{h}\pm0.23$	$120^{c}\pm0.22$	$14^{i}\pm0.48$	$86^{ef}\pm0.77$	$1.9^{h}\pm0.008$	$1.3^{fg}\pm0.098$	$2^{g}\pm0.008$	$1.6^{h}\pm0.015$
14	Sepidan	$9.2^{d-f}\pm0.09$	$64^{b}\pm1.44$	$9.1^{b-d}\pm0.06$	$41^{b}\pm1$	$38^{b}\pm1$	$139^{a}\pm1.5$	$29^{cd}\pm0.92$	$119^{a}\pm0.34$	$3.2^{c-e}\pm0.017$	$2.2^{bc}\pm0.108$	$5.5^{b}\pm0.199$	$2.3^{fg}\pm0.114$
15	Soltanieh	$11^{b-d}\pm0.14$	46ef±1.15	$7^{fg}\pm0.04$	$35^{c}\pm0.84$	$29^{de}\pm0.86$	$128^{b}\pm1.32$	$24^{d-f}\pm0.86$	$97^{c}\pm0.94$	$3.8^{a-c}\pm0.117$	$2^{b-d}\pm0.096$	$4.2^{d}\pm0.122$	$2.3^{fg}\pm0.108$
16	Taft	$15^{a}\pm0.18$	43f±1.45	$9.8^{ab}\pm0.12$	$35^{c}\pm0.52$	$49^{a}\pm1.32$	$110^{d}\pm1.15$	$25^{c-f}\pm1$	$107^{b}\pm1$	$4.1^{ab}\pm0.114$	$3^{a}\pm0.119$	$8.1^{a}\pm0.102$	$4^{a}\pm0.182$

According to the analysis of variance that triple effects of Azotobacter, salinity and accessions showed significant difference, slice method used for mean comparisons. Mean values with the same letters within a column are not significantly different ($p<0.05$), Tukey test. Means ± standard error (SE).

Table S4. Effect of Azotobacter and salinity stress interaction on measured metabolites of 16 Iranian licorice accessions.

		Traits											
		Root Iron (ppm)				Root Potassium (ppm)				Root Phosphorus (ppm)			
		No-Bacterial treatment		Bacterial treatment		No-Bacterial treatment		Bacterial treatment		No-Bacterial treatment		Bacterial treatment	
No.	Accessions	No-Salt treatment	Salt treatment	No-Salt treatment	Salt treatment	No-Salt treatment	Salt treatment	No-Salt treatment	Salt treatment	No-Salt treatment	Salt treatment	No-Salt treatment	Salt treatment
1	Baft	$2.9^{de}\pm0.05$	$2.3^{cd}\pm0.06$	$5.5^{c}\pm0.11$	$2.7^{ef}\pm0.08$	$125^{a}\pm2.04$	$107^{b}\pm1.12$	$128^{b}\pm1.64$	$110^{bc}\pm1.16$	$41^{d}\pm1.21$	$30^{gh}\pm0.58$	$43^{de}\pm0.5$	$37^{de}\pm0.98$
2	Bajgah	$2.4^{e}\pm0.1$	$2.3^{cd}\pm0.07$	$2.5^{ij}\pm0.15$	$2.4^{f}\pm0.04$	$106^{c-e}\pm1.03$	$97^{d}\pm0.96$	$114^{cd}\pm1$	$102^{d}\pm0.82$	$36^{e}\pm1.04$	$29^{gh}\pm1.01$	$37^{f}\pm1$	$36^{ef}\pm0.44$
3	Bardsir	$4.6^{b}\pm0.07$	$3.3^{b}\pm0.1$	$6.1^{b}\pm0.18$	$4.2^{bc}\pm0.1$	$126^{a}\pm2.1$	$116^{a}\pm2.04$	$137^{a}\pm2.08$	$123^{a}\pm2.12$	$36^{e}\pm1.01$	$35^{f}\pm1.18$	$43^{de}\pm0.94$	$36^{ef}\pm0.5$
4	Darab	$4.8^{b}\pm0.02$	$2.6^{c}\pm0.02$	$5.2^{cd}\pm0.12$	$3.3^{d}\pm0.15$	$105^{c-e}\pm0.54$	$103^{bc}\pm1.15$	$110^{de}\pm0.98$	$105^{cd}\pm0.56$	$51^{a}\pm1.83$	$48^{a}\pm1$	$62^{a}\pm1.44$	$50^{a}\pm1.15$
5	Eghlid	$5.8^{a}\pm0.15$	$3.2^{b}\pm0.05$	$6.7^{a}\pm0.1$	$4.4^{ab}\pm0.18$	$89^{g}\pm0.9$	$81^{gh}\pm0.57$	$91^{g}\pm1.13$	$87^{f-h}\pm0.08$	$44^{cd}\pm1.08$	$41^{b-d}\pm0.54$	$47^{d}\pm0.57$	$42^{bc}\pm1$
6	Ilam	$1.7^{f}\pm0.09$	$1.4^{e}\pm0.01$	$2.1^{j}\pm0.05$	$1.5^{g}\pm0.06$	$107^{cd}\pm1.02$	$78^{h}\pm0.88$	$114^{cd}\pm0.86$	$85^{gh}\pm0.58$	$29^{f}\pm0.57$	$26^{h}\pm0.18$	$31^{g}\pm0.64$	$29^{g}\pm0.08$
7	Kashmar	$3^{c}\pm0.11$	$2.6^{c}\pm0.08$	$3.7^{g}\pm0.16$	$2.6^{f}\pm0.1$	$109^{bc}\pm0.98$	$99^{cd}\pm1$	$112^{cd}\pm0.42$	$109^{bc}\pm0.98$	$41^{d}\pm0.77$	$33^{fg}\pm0.98$	$42^{e}\pm1.15$	$39^{c-e}\pm0.66$
8	Kermanshah	$2.8^{de}\pm0.04$	$2.6^{c}\pm0.05$	$3.2^{h}\pm0.07$	$2.7^{ef}\pm0.05$	$103^{de}\pm0.66$	$86^{f}\pm0.48$	$106^{ef}\pm1.1$	$92^{ef}\pm1$	$41^{d}\pm0.07$	$40^{c-e}\pm1.15$	$43^{de}\pm0.86$	$40^{c-e}\pm0.12$
9	Mahabad	$4.4^{b}\pm0.1$	$3.2^{b}\pm0.1$	$4.5^{ef}\pm0.14$	$3.9^{c}\pm0.07$	$113^{b}\pm1.12$	$99^{cd}\pm1.15$	$128^{b}\pm1.58$	$107^{b-d}\pm1.04$	$49^{ab}\pm1.15$	$45^{ad}\pm1.1$	$53^{c}\pm1.24$	$47^{a}\pm0.24$
10	Marvast	$2.6^{de}\pm0.08$	$2.4^{cd}\pm0.04$	$2.9^{hi}\pm0.08$	$2.5^{f}\pm0.1$	$93^{fg}\pm0.52$	$79^{h}\pm0.64$	$95^{g}\pm1.15$	$89^{ef}\pm0.68$	$35^{e}\pm0.58$	$26^{h}\pm0.68$	$45^{de}\pm1$	$32^{fg}\pm0.31$
11	Meshkinshahr	$4.7^{b}\pm0.01$	$2.5^{c}\pm0.01$	$5^{d}\pm0.16$	$3.2^{d}\pm0.16$	$90^{fg}\pm0.58$	$85^{fg}\pm0.74$	$95^{g}\pm0.94$	$85^{gh}\pm0.08$	$53^{a}\pm1.19$	$45^{ab}\pm1.14$	$56^{bc}\pm0.5$	$48^{a}\pm1.08$
12	Piranshahr	$2.4^{e}\pm0.05$	$2^{d}\pm0.03$	$3.1^{h}\pm0.04$	$2.4^{f}\pm0.09$	$90^{fg}\pm0.78$	$71^{i}\pm0.92$	$95^{g}\pm0.77$	$83^{h}\pm0.04$	$45^{b-d}\pm1.14$	$40^{c-e}\pm0.66$	$46^{de}\pm0.72$	$42^{bc}\pm1$
13	Rabt	$2.9^{de}\pm0.02$	$2.4^{cd}\pm0.05$	$4.1^{fg}\pm0.17$	$2.6^{f}\pm0.02$	$94^{f}\pm1.15$	$86^{f}\pm1.12$	$104^{f}\pm1.05$	$92^{ef}\pm1.1$	$51^{a}\pm0.56$	$44^{a-c}\pm1.24$	$60^{ab}\pm1.54$	$46^{ab}\pm1.13$
14	Sepidan	$3.6^{c}\pm0.12$	$2.3^{cd}\pm0.07$	$4.9^{de}\pm0.1$	$3.1^{de}\pm0.03$	$125^{a}\pm2$	$105^{b}\pm1.19$	$128^{b}\pm1.15$	$106^{b-d}\pm1.18$	$41^{d}\pm1$	$36^{ef}\pm0.92$	$44^{de}\pm1.15$	$37^{de}\pm0.96$
15	Soltanieh	$2.7^{de}\pm0.04$	$2.3^{cd}\pm0.09$	$3.2^{h}\pm0.05$	$2.5^{f}\pm0.07$	$101^{e}\pm0.48$	$92^{e}\pm0.77$	$116^{c}\pm1.08$	$93^{e}\pm0.42$	$42^{cd}\pm0.86$	$35^{f}\pm0.88$	$46^{de}\pm0.82$	$39^{c-e}\pm0.57$
16	Taft	$4.9^{b}\pm0.06$	$3.9^{a}\pm0.1$	$5.6^{c}\pm0.07$	$4.7^{a}\pm0.12$	$124^{a}\pm1$	$107^{b}\pm1.14$	$127^{b}\pm1$	$111^{b}\pm1.15$	$46^{bc}\pm1.15$	$37^{d-f}\pm0.46$	$53^{c}\pm1.08$	$41^{cd}\pm0.72$

According to the analysis of variance that triple effects of Azotobacter, salinity and accessions showed significant difference, slice method used for mean comparisons. Mean values with the same letters within a column are not significantly different (p<0.05), Tukey test. Means ± standard error (SE).

Table S5. Eigenvalues and cumulative variance for factors obtained from the principal component analysis (PCA) based on morphophysiological and phytochemical traits for the studied Accessions of *G.glabra*

Variable	Component								
	1	2	3	4	5	6	7	8	9
Root Fresh Weight	0.35	0.03	-0.11	0.02	-0.28	-0.29	-0.03	0.02	-0.15
Root Dry Weight	0.35	0.06	-0.14	-0.03	-0.24	-0.32	-0.00	-0.05	-0.08
Glycyrrhizic acid	0.22	0.06	0.29	-0.15	0.32	-0.00	0.25	0.02	0.19
Glabridin	-0.14	0.00	-0.21	-0.27	0.04	0.05	-0.48	-0.42	0.21
Total Protein	-0.08	0.34	0.13	0.09	-0.19	0.11	-0.09	-0.22	-0.18
Total Starch	0.11	-0.29	0.04	0.31	-0.00	0.05	-0.18	-0.22	-0.39
Rutin	-0.12	0.16	0.39	0.05	0.18	0.00	0.1	-0.38	0.06
Total Flavonoid	-0.07	-0.09	-0.00	0.06	0.34	-0.55	-0.07	-0.17	0.04
Total Flavonol	-0.2	0.00	-0.25	0.17	0.27	-0.19	0.31	0.12	-0.26
Total Sugar	-0.32	-0.02	-0.24	-0.18	0.03	0.13	0.03	-0.19	-0.19
Total Phenol	-0.01	-0.35	-0.02	0.06	-0.01	-0.13	0.44	-0.34	0.19
Total Tannin	-0.18	-0.16	-0.35	0.13	-0.12	-0.26	-0.01	-0.04	0.28
Root K	0.09	0.15	-0.07	0.02	0.56	-0.02	-0.17	-0.03	-0.23
Root P	-0.15	0.07	0.11	0.46	-0.06	0.11	0.1	0.06	0.23
Root Fe	0.13	0.06	-0.1	0.46	0.16	0.09	-0.17	0.02	0.27
Root Cl	0.12	-0.41	0.02	-0.1	0.19	0.07	-0.03	0.14	-0.29
Root Na	0.11	-0.29	-0.19	0.22	0.11	0.26	-0.26	0.03	0.18
Leaf K	0.28	0.079	-0.23	-0.28	0.2	0.21	0.05	0.07	0.12
Leaf P	0.22	0.047	-0.17	0.25	-0.02	0.32	0.25	-0.33	-0.27
Leaf N	-0.08	-0.33	0.34	0.04	-0.03	0.02	-0.18	0.31	-0.02
Leaf Fe	0.19	0.34	0.02	0.21	0.08	-0.23	-0.2	0.08	-0.00
Leaf Cl	0.17	-0.23	0.36	-0.04	-0.06	-0.12	-0.17	-0.3	-0.02
Leaf Na	0.41	-0.08	-0.06	-0.06	0.04	0.11	0.14	-0.13	0.25
Eigenvalue	4.59	3.82	3.18	2.96	2.22	1.71	1.16	0.91	0.73
% Of Variance	20	16	13.8	12.9	9.7	7.4	5.1	4	3.2
Cumulative %	20	36.6	50.4	63.3	73	80.5	85.5	89.5	92.7

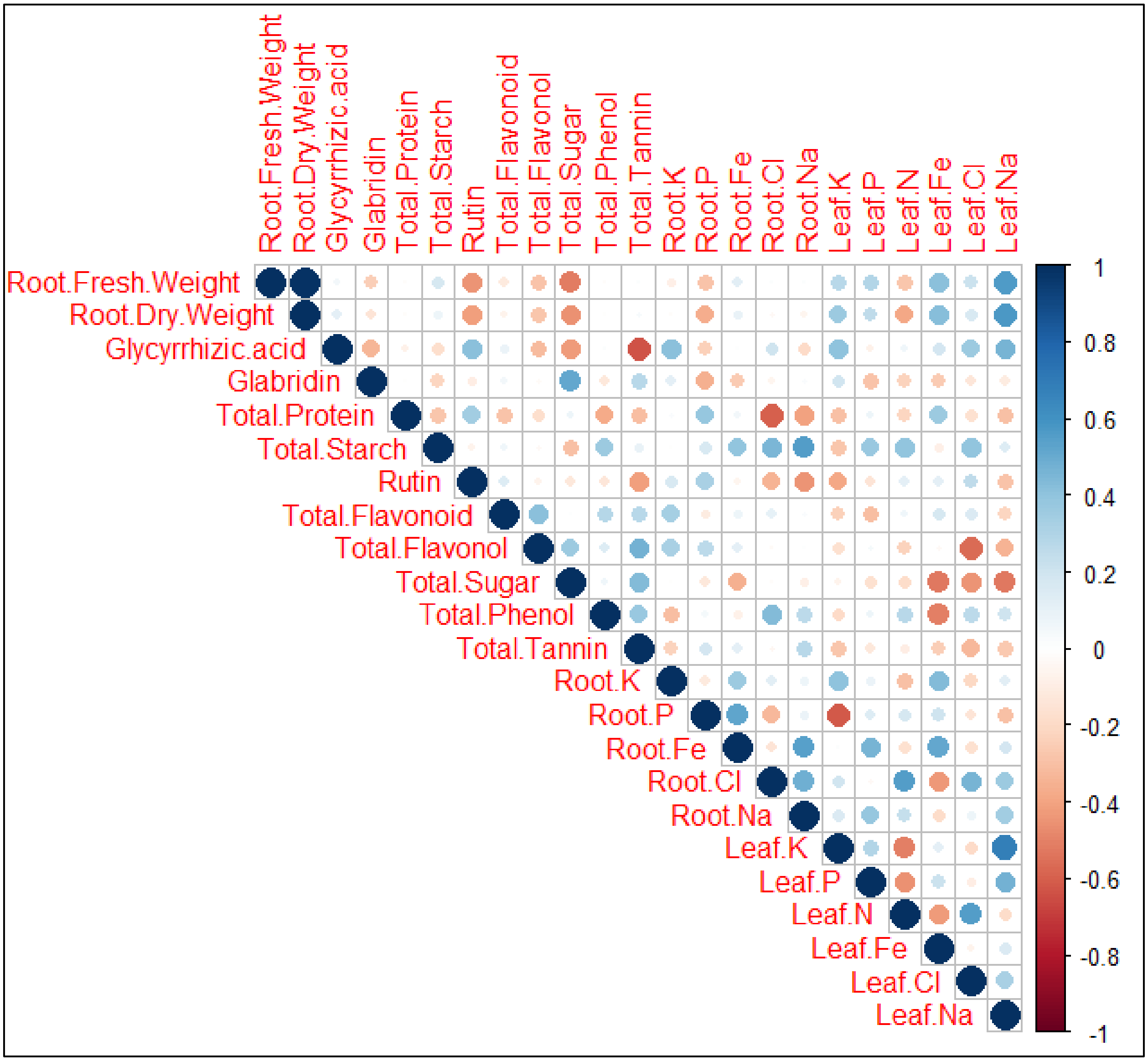

Fig S1. Corrplot between various measured parameters.
